# Combination Cyclophosphamide and Rituximab to Minimize Glucocorticoid Use in Antineutrophil Cytoplasm Antibody–Associated Vasculitis

**DOI:** 10.1016/j.ekir.2025.04.030

**Published:** 2025-04-22

**Authors:** Tania Salehi, Gavin B. Chapman, Tariq E. Farrah, Fiona A. Chapman, Dan Pugh, Robert W. Hunter, Neeraj Dhaun

**Affiliations:** 1 Department of Renal Medicine, Royal Infirmary of Edinburgh, Edinburgh, UK; 2 Central and Northern Adelaide Renal and Transplantation Service, Adelaide, South Australia, Australia; 3 Edinburgh Kidney Research Group, University/BHF Centre for Cardiovascular Science, The Queen's Medical Research Institute, University of Edinburgh, Edinburgh, UK

**Keywords:** ANCA vasculitis, cyclophosphamide, glucocorticoids, induction treatment, rituximab

## Abstract

**Introduction:**

The optimal duration of immunosuppressive therapy for antineutrophil cytoplasm antibody (ANCA)-associated vasculitis (AAV) is uncertain. Glucocorticoids are a mainstay of treatment but are associated with significant morbidity. Here, we describe outcomes of a cohort of patients treated with combination cyclophosphamide and rituximab induction alongside a rapidly tapering oral-only glucocorticoid regimen.

**Methods:**

In this retrospective, observational, cohort study, we identified patients presenting with AAV between 2011 and 2023 treated with combination cyclophosphamide and rituximab induction therapy. We analyzed biochemical, histologic, and outcome data, including time-to-remission and relapse rate. A subgroup analysis compared outcomes based on glucocorticoid duration.

**Results:**

A total of 112 patients with active AAV treated with combination cyclophosphamide and rituximab were identified (median age: 67 years; 85% kidney involvement; baseline estimated glomerular filtration rate [eGFR] 24 ml/min per 1.73 m^2^). Of the patients, 96% achieved remission; median time-to-remission was 77 (interquartile range [IQR]: 64–92) days. All patients demonstrated biochemical and histologic improvement following treatment. Five patients (5%) experienced a disease relapse over 2.9 (IQR: 1.7–4.3) years follow-up. The cumulative glucocorticoid dose was 1780 (IQR: 1141–2935) mg with median duration of 12.5 (IQR: 8.0–39.0) weeks. Patients treated with oral glucocorticoids for > 12 weeks received a higher cumulative dose (2935 vs. 1133 mg; *P* < 0.001) with a trend toward more serious infections (21% vs. 7%; *P* = 0.06) than those treated for *≤* 12 weeks with no differences in disease remission (100% vs. 91%; *P* = 0.07) or relapse (9% vs. 0%; *P* = 0.07) rates.

**Conclusion:**

Early withdrawal of oral glucocorticoid therapy in patients with severe AAV treated with combination cyclophosphamide and rituximab induction immunosuppression is safe and effective and may reduce morbidity, in particular serious infections.

## Graphical abstract

**Figure:**
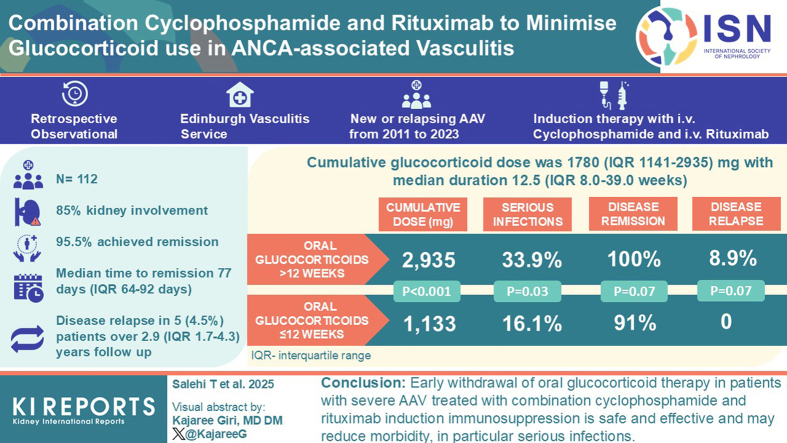

AAV comprises a group of autoimmune disorders characterized by inflammation and injury to small and medium-sized blood vessels in any tissue or organ, with the upper and lower respiratory tract and kidneys commonly involved.^1^ Advances in immunosuppressive therapy have transformed AAV from an acute, life-threatening illness to a chronic, relapsing condition where balancing accrual of organ damage against treatment-related toxicity remains a key challenge. Current international guidelines for the management of AAV recognize the heterogenous nature of the disease and the range of immunosuppressive therapies available but are limited in their guidance on treatment duration and tailoring to specific clinical phenotypes.^2^, ^3^, ^4^

Glucocorticoids are the mainstay of treatment for AAV in combination with either cyclophosphamide or rituximab.^2^, ^3^, ^4^ However, glucocorticoid use is often complicated by numerous side-effects.^5^ In the short-term, infection is the main treatment-related complication.^6^ In the longer term, cardiovascular disease and osteoporosis are important morbidities.^7^ Thus, recent studies have focused on glucocorticoid-sparing regimens that treat AAV effectively but reduce complications.^8^ The landmark PEXIVAS study defined a new “reduced dose” glucocorticoid tapering regimen following induction therapy with i.v. methylprednisolone and either cyclophosphamide or rituximab.^9^ Although now accepted as standard-of-care,^2^, ^3^, ^4^ this regimen still represents a relatively high cumulative glucocorticoid burden and the use of i.v. methylprednisolone may be associated with serious infections and incident diabetes mellitus, irrespective of total glucocorticoid dose.^10^ There remains uncertainty about the optimal duration of glucocorticoid therapy. Commonly, glucocorticoids are prescribed for periods exceeding 12 months to prevent disease relapse. This is in the presence of conflicting data demonstrating benefit^11^^,^^12^ and with good evidence that this increases the risk of long-term toxicity.^13^^,^^14^

Combination cyclophosphamide-rituximab induction, with i.v. methylprednisolone followed by a rapid oral glucocorticoid taper, was shown to be effective and safe in an observational study of patients with AAV causing glomerulonephritis.^15^ In this study, first-line maintenance immunosuppression was with azathioprine. Here, we report the long-term outcomes of a larger cohort of patients with AAV, including those without kidney involvement, who received combination cyclophosphamide-rituximab induction but no i.v. glucocorticoid, alongside a rapidly reducing and withdrawing oral glucocorticoid taper and maintenance immunosuppression with rituximab.

## Methods

### Patient Population

We conducted a study of patients presenting with active AAV to the Edinburgh Vasculitis Service. We screened all patients presenting with new or relapsing AAV from January 2011 to December 2023 and included those who received induction therapy with a combination of i.v. cyclophosphamide and i.v. rituximab. The diagnosis of active disease was made by a Consultant Vasculitis Physician, in line with the 2012 Chapel Hill Consensus Classification,^16^ and with supporting clinical, histologic, laboratory, and radiological evidence of active AAV and a requirement for systemic immunosuppressive therapy.

We excluded patients who were positive for both ANCA and glomerular basement membrane antibodies, those who had received an alternative induction regimen (including cyclophosphamide or rituximab monotherapy), as well as those who had received i.v. methylprednisolone at presentation. Kidney involvement was defined by either (i) kidney biopsy proven pauci-immune glomerulonephritis; or, in patients where biopsy was contraindicated or not feasible, acute kidney injury and/or the presence of hematuria and/or proteinuria without reasonable alternative cause.

### Data Collection

We analyzed routinely collected clinical and laboratory data from the electronic patient record and from SQL-based database queries. All laboratory data from the vasculitis clinic visits and any other health care encounters in primary and secondary care within our health board were recorded. These included ANCA serology and titer, Birmingham Vasculitis Activity Score (BVAS) Version 3.0,^17^ high-sensitivity C-reactive protein, serum creatinine, eGFR (calculated using the Chronic Kidney Disease Epidemiology Collaboration 2021 equation^18^), and urinary protein-to-creatinine ratio. For patients in whom the BVAS was not documented in the notes, this was calculated retrospectively. For those patients undergoing presentation and interval kidney biopsies, we collected information related to glomeruli (total number and number of necrotizing lesions, cellular crescents, fibrocellular crescents, and global glomerulosclerosis), interstitial fibrosis, and interstitial nephritis. Renal risk classifications were determined for these patients in accordance with the criteria outlined by Berden *et al.*^19^ and Bate *et al.*^20^ for both initial and interval kidney biopsies.^19^^,^^20^

We recorded the length of hospital stay during the index admission, time to disease remission, major disease relapse history, and maintenance immunosuppression. We quantified glucocorticoid exposure as the total number of weeks on treatment and the cumulative dose of oral prednisolone in mg. Patients were given i.v. hydrocortisone 200 mg before rituximab therapy to minimize the risk of an infusion reaction. This is equivalent to approximately 50 mg oral prednisolone. This was included in the calculated cumulative glucocorticoid dose. Disease remission was defined as a BVAS score of 0. Disease relapse was defined as a change in symptoms or signs along with a new, worsening of, or recurrence of any item on BVAS that, after a period of complete remission and appropriate investigation, was likely attributable to vasculitis disease activity and required an escalation in immunosuppressive treatment.

### Disease Treatment

All patients included received a maximum of 2 doses of i.v. cyclophosphamide and 2 doses of i.v. rituximab, at least 2 weeks apart. Cyclophosphamide doses were adjusted according to age and kidney function, and no individual dose was > 1000 mg. Patients commenced oral prednisolone at diagnosis of active disease. In all patients, maintenance immunosuppression was with rituximab 500 to 1000 mg given at fixed 6- to 12-monthly intervals; rituximab dosing was not based on peripheral B-cell counts (Supplementary Figure S1). Decisions about tapering and withdrawal of oral glucocorticoid therapy were made on an individual patient-basis by their vasculitis physician and were not based on any single clinical parameter.

All patients received *Pneumocystis jirovecii* prophylaxis with trimethoprim/sulphamethoxazole. Patients who developed adverse reactions underwent desensitization using our in-house protocol (Supplementary Table S1). In addition, all patients received prophylaxis against peptic ulcer disease and calcium/vitamin D supplementation for the duration of glucocorticoid therapy.

### Disease Outcomes

We determined time-to-remission and time-to-relapse. We also recorded key laboratory results at baseline and at months 3, 6, and 12. For patients who were receiving kidney replacement therapy at these time points, serum creatinine and eGFR were recorded as the result immediately before commencing the therapy . For ANCA serology, if the ANCA titer was above the upper limit of the assay, it was recorded as the upper limit of detection plus 1 (e.g., if proteinase 3 titer was > 200 IU/ml, then this was recorded as 201). We captured adverse events, which included new kidney failure (eGFR ≤15 ml/min per 1.73 m^2^ for > 90 days having been > 15 ml/min per 1.73 m^2^ at disease presentation or new receipt of kidney replacement therapy), rate of hospitalization for infections, fragility fractures, cardiovascular disease (defined as ischemic heart disease, stroke, heart failure, arrhythmia, or venous thromboembolism), cataracts, malignancy, significant weight gain (defined as ≥ 5% from baseline^21^), hypogammaglobulinemia (classified as moderate (IgG: 3.0–4.9 g/l) or severe (IgG ≤ 2.9 g/l), respectively^22^), lymphopenia (lymphocyte count < 1 × 10^9^/l), neutropenia (neutrophil count < 1.5 × 10^9^/l), and death. Total follow-up duration was from time of disease presentation to either date of death or study censor date (June 30, 2024). Because this was a retrospective analysis, it met the criteria for a service evaluation study and so did not require approval from a research ethics committee.

### Statistical Analysis

Continuous variables were reported as group means ± SD if normally distributed or as median ± IQR, if not. The average differences in baseline characteristics between cohorts receiving < or > 12 weeks of glucocorticoids were compared using 2-tailed *t* tests for normally distributed continuous variables, Mann-Whittney-U tests for nonnormally distributed continuous variables and χ^2^ testing for categorical variables. Data missingness was handled by performing complete case analyses. Survival (Kaplan-Meier) analysis was performed to compare time-to-remission between the 2 groups. Data were analyzed in R (version 4.3.2).^23^ The Kaplan-Meier survival analysis was performed using the Survival (v3.4-0) and Survminer (version 0.4.9) packages; groups were compared using a log-rank test. Statistical significance was defined as a 2-tailed *P* < 0.05.

## Results

### Baseline Characteristics

Between 2011 and 2023, a total of 112 patients with active AAV received combination induction treatment with cyclophosphamide and rituximab (Supplementary Figure S1). Of these, 88% (98/112) were first presentations; 12% (14/112) had relapsing disease. Baseline characteristics of the study population are presented in Table 1.

**Table 1:** Demographic, clinical and laboratory characteristics of patients at baseline

Characteristics	Cohort (*N* = 112)
Age, yrs	67 (56–76)
Male sex, *n* (%)	64 (57)
Disease status	
First presentation	98 (88)
Relapsed disease	14 (12)
Organ involvement	
ENT	23 (21)
Respiratory	68 (61)
Pulmonary hemorrhage	15 (13)
Renal	95 (85)
eGFR < 15 ml/min per 1.73 m^2^ or requiring dialysis	34 (30)
Nervous system	25 (22)
Ocular	13 (12)
Cutaneous	9 (8)
Rheumatologic	15 (13)
AAV phenotype	
GPA	48 (43)
MPA	58 (52)
EGPA	6 (5)
ANCA serology	
PR3	46 (41)
MPO	47 (42)
Dual PR3/MPO positive	5 (5)
Negative	14 (12)
Baseline biochemistry	
Creatinine, μmol/l	183 (98–349)
eGFR, ml/min per 1.73 m^2^	28 (14–70)
uPCR, mg/mmol	101 (36–198)
CRP, mg/l	44 (8–101)
Disease activity (BVAS)	15 (11–19)
Kidney biopsy (*n* = 75)	
Berden class	
Focal	22 (29)
Crescentic	18 (24)
Mixed	29 (39)
Sclerotic	6 (8)
AKRiS	
Low	40 (53)
Moderate	27 (36)
High	7 (9)
Very high	1 (1)
Treatment	
Induction therapy	
Cyclophosphamide, g	1.0 (1.0–2.0)
Rituximab, g	2.0 (2.0–2.0)
Plasma exchange, *n* (%)	8 (7)
Maintenance agent	
Rituximab	100 (89)
Azathioprine	2 (2)
None	10 (9)
Oral prednisolone	
Cumulative dose, g	1.8 (1.1–2.9)
Duration, wks	12.5 (8.0–39.0)

AAV, ANCA-associated vasculitis; AKRiS, ANCA Kidney Risk Score; ANCA, antineutrophil cytoplasm antibody; BVAS, Birmingham Vasculitis Activity Score; CRP, C-reactive protein; eGFR, estimated glomerular filtration rate; EGPA, eosinophilic granulomatosis with polyangiitis; ENT, ear, nose and throat; GPA, granulomatosis with polyangiitis; MPA, microscopic polyangiitis; MPO, myeloperoxidase; PR3, proteinase 3; uPCR, urinary protein-to-creatinine ratio.

Data are presented as median (interquartile range) or number of patients (%).

Missing data: uPCR = 12; CRP = 2.

The cohort was representative of a typical AAV population. The median age was 67 (56–76) years with a slight male predominance (64/112 [57%]). Median BVAS at disease presentation was 15 (11–19). Most patients had kidney involvement (95/112 [85%]). In these patients, baseline serum creatinine was 225 (108–380) μmol/l and eGFR 24 (13–52) ml/min per 1.73 m^2^; proteinuria was approximately 1.1 g/d (urinary protein-to-creatinine ratio: 112 (59–210) mg/mmol). Thirty-four of these 95 patients (36%) presented with an eGFR < 15 ml/min per 1.73 m^2^, of whom 4 were dialysis-dependent. Kidney biopsies were performed in 75 of 95 patients with kidney involvement (79%).

Most patients were classified as having either microscopic polyangiitis (58/112 [52%]) or granulomatosis with polyangiitis (GPA) (48/112 [43%]); 6 patients (5%) had eosinophilic GPA. The representation of myeloperoxidase- and proteinase 3–ANCA was similar across the cohort (47/112 [42%] vs. 46/112 [41%], respectively). Baseline characteristics of patients by ANCA serotype are summarized in Supplementary Table S2.

### Disease Treatment

One hundred eleven of 112 patients received disease induction immunosuppression as described (Supplementary Figure S2). The remaining patients received 4 doses of i.v. cyclophosphamide (total dose delivered: 4000 mg). Fifty-seven patients (51%) received induction treatment in an outpatient setting. For the remaining 55 patients (49%), the median length of hospital stay was 9.5 (5.1–16.1) days. One hundred patients (89%) received maintenance immunosuppression with rituximab. Of the remaining 12 patients, 6 died before commencing maintenance therapy, 2 presented before 2013 and received azathioprine maintenance therapy, 3 were frail and received no further immunosuppression following induction, and 1 patient remained on renal replacement therapy at 6 months and chose not to have further immunosuppression.

### Disease Outcomes

**Figure 1:**
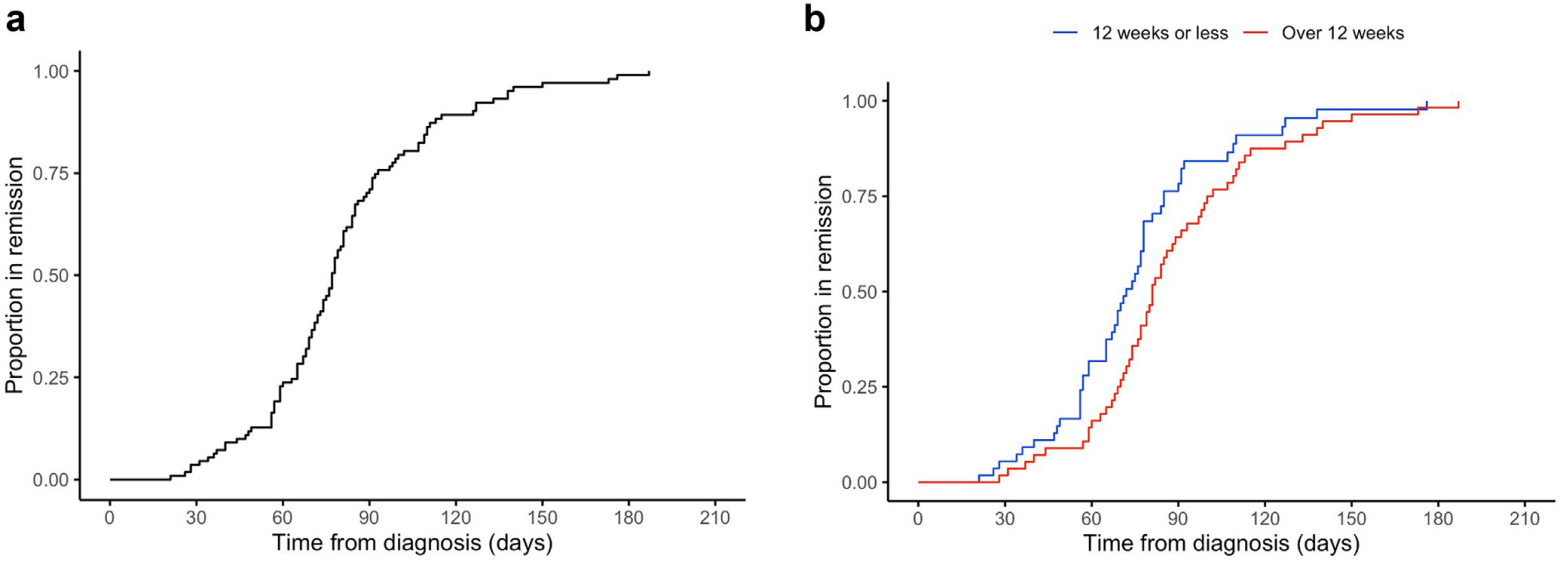
(a) Cumulative incidence curve for time to remission in whole cohort treated with combination cyclophosphamide and rituximab and (b) stratified by duration of glucocorticoid therapy. Log-rank test to compare time to remission between groups, *P* = 0.045.

One hundred seven patients (96%) achieved treatment-induced disease remission (median time-to-remission 77 [IQR: 64–92] days) (Figure 1a). The remaining 5 patients (4%) died with active vasculitis (Supplementary Table S3). Changes in biochemical and immunologic laboratory measures following the initiation of induction therapy are illustrated in Supplementary Figure S3. Biomarkers of disease activity, including ANCA titer and C-reactive protein, demonstrated early and sustained improvements.

**Figure 2:**
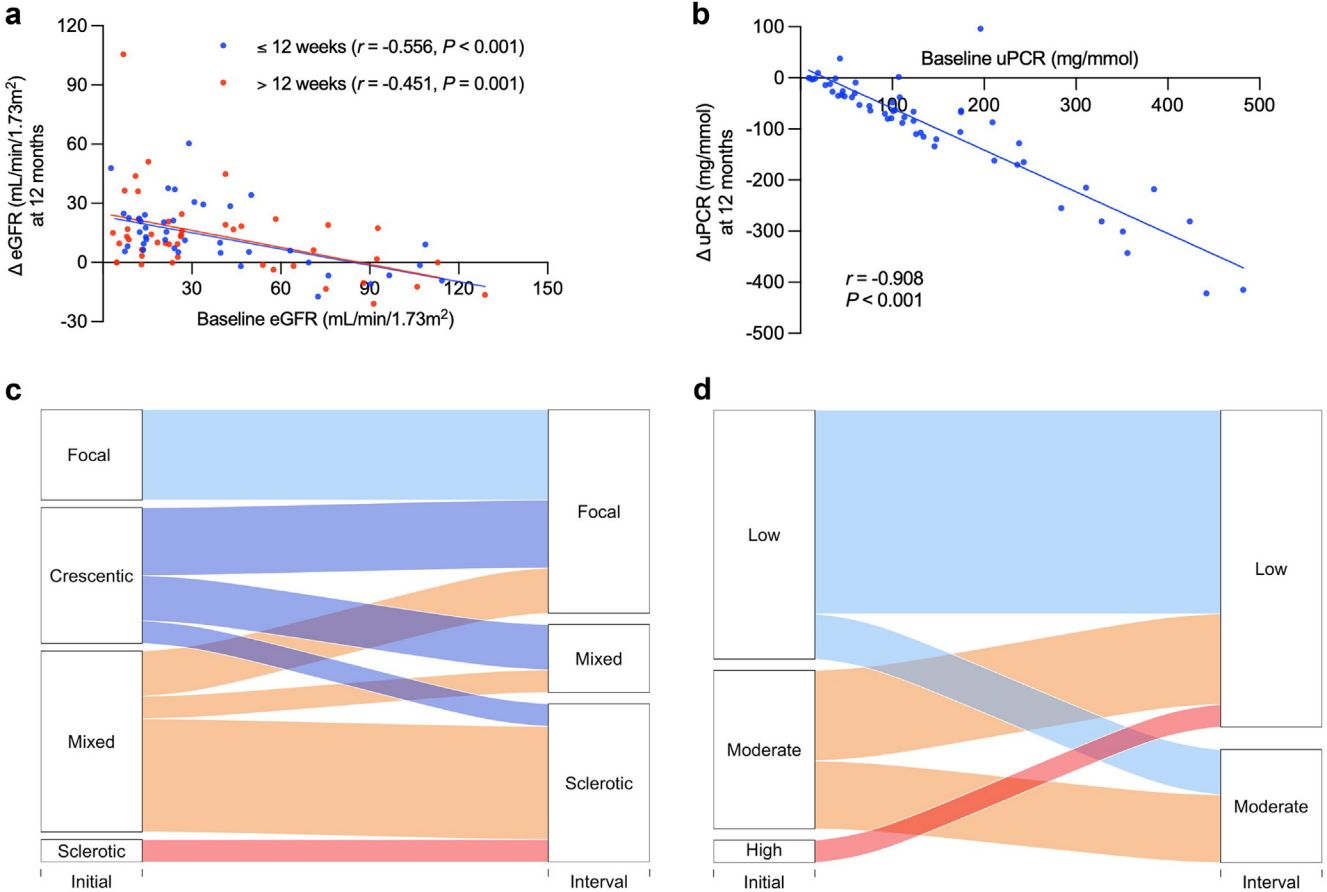
(a) Kidney response to treatment. Scatter plots of individual change in eGFR at 12 months versus baseline eGFR, stratified by duration of glucocorticoid therapy, and (b) individual change in uPCR at 12 months versus baseline uPCR for the whole cohort. *r* values are Pearson coefficients. Alluvial plots demonstrating change in AAV classification from initial biopsy to interval biopsy (*n =* 19) for (c) Berden classification, and (d) ANCA Kidney Risk Score. AAV, antineutrophil cytoplasm antibody-associated vasculitis; ANCA, antineutrophil cytoplasm antibody; eGFR, estimated glomerular filtration rate; uPCR, urinary protein-to-creatinine ratio.

In those with renal involvement, kidney function improved over the 12 months after diagnosis (creatinine: 225 [IQR: 108–380] μmol/l at diagnosis vs. 141 [IQR: 95–190] μmol/l at 12 months, *P* < 0.001; eGFR: 24 [IQR: 13–52] ml/min per 1.73 m^2^ at diagnosis vs. 45 [IQR: 31–69] ml/min per 1.73 m^2^ at 12 months, *P* < 0.001; urinary protein-to-creatinine ratio: 112 [IQR 59–210] mg/mmol at diagnosis vs. 33 [IQR: 17–68] mg/mmol at 12 months, *P* < 0.001). Those with more severe kidney impairment at diagnosis had a greater improvement in eGFR and proteinuria following treatment than those with more preserved kidney function (Figure 2a and b).

Of the 75 patients who had a kidney biopsy at diagnosis, 19 (25%) had an interval kidney biopsy at a median time interval of 143 (117–186) days. At presentation, these patients had a broad range of renal risk based on both the Berden classification and ANCA Kidney Risk Score (Figures 2c and d). We evaluated how this risk changed in response to treatment. In summary, for both the Berden classification and ANCA Kidney Risk Score, interval biopsies were in keeping with a lower risk for future kidney failure than presentation biopsies.

Over a median follow-up of 2.9 (1.7–4.3) years, 5 relapse episodes occurred in 5 patients (4%) with median time-to-relapse of 3.1 (2.2–3.2) years. Seventeen patients (15%) died during follow-up with median time-to-death of 1.8 (0.3–4.4) years. Overall, patient survival at 1, 3, and 5 years was 95% (106/112), 90% (101/112), and 88% (98/112), respectively.

### Glucocorticoid Exposure

Overall, patients received 12.5 (8.0–39.0) weeks of prednisolone with a cumulative dose of 1780 (1141–2935) mg. Those with a first presentation of disease received 12.0 (8.0–39.5) weeks of treatment at a cumulative dose of 1780 (1159–2935) mg compared with 27.5 (7.8–34.8) weeks of prednisolone at a cumulative dose of 1990 (1198–2743) mg in patients treated for relapsing disease (*P* = 0.86).

### Greater Than 12 Weeks Versus ≤12 Weeks of Glucocorticoid Therapy

Given that the median duration of glucocorticoid exposure in our cohort was approximately 12 weeks, we examined outcomes in patients receiving > or ≤ 12 weeks of glucocorticoid therapy. Baseline characteristics, treatment details, remission and relapse rates, and adverse events of patients receiving glucocorticoid therapy for > 12 weeks versus those receiving it for 12 weeks or less are summarized in Table 2. In brief, the 2 groups were well-matched. However, there was a lower prevalence of eosinophilic GPA in patients receiving ≤ 12 weeks of glucocorticoid therapy (0/56 [0%] vs. 6/56 [11%], *P* = 0.04). In addition, baseline C-reactive protein was lower in this group than in those receiving > 12 weeks of glucocorticoids (24 [6–63] vs. 67 [15–123] mg/l, *P* < 0.01). Those patients treated with oral glucocorticoid therapy for > 12 weeks received a cumulative dose of 2935 (2262–3390) mg compared with 1133 (861–1570) mg in patients treated for ≤ 12 weeks (*P* < 0.001).

**Table 2:** Glucocorticoid therapy ≤ 12 versus > 12 weeks

Characteristics	≤ 12 wks (n = 56)	> 12 wks (n = 56)	*P*-value
Age, yrs	68 (61–76)	65 (53–75)	0.20
Male sex, *n* (%)	31 (55)	33 (59)	0.85
Baseline weight, kg	72.5 ± 16.6	74.7 ± 16.1	0.59
Organ involvement			
ENT	10 (18)	13 (23)	0.64
Respiratory	32 (57)	36 (64)	0.56
Pulmonary hemorrhage	6 (11)	9 (16)	0.58
Renal	48 (86)	47 (84)	1.00
eGFR < 15 ml/min per 1.73 m^2^ or requiring dialysis	18 (32)	16 (29)	0.84
Nervous system	11 (20)	14 (25)	0.65
Ocular	5 (9)	8 (14)	0.56
Cutaneous	5 (9)	4 (7)	1.00
Rheumatologic	8 (14)	7 (13)	1.00
ANCA serology			
PR3	23 (41)	23 (41)	0.11
MPO	28 (50)	19 (34)	
Dual positive	3 (5)	2 (4)	
Negative	2 (4)	12 (21)	
AAV phenotype			
GPA	25 (45)	23 (41)	<0.05
MPA	31 (55)	27 (48)	
EGPA	0	6 (11)	
Baseline biochemistry			
Creatinine, μmol/l	183 (107–342)	201 (95–358)	0.96
eGFR, ml/min per 1.73 m^2^	30 (14–59)	27 (13–75)	0.79
uPCR, mg/mmol	86 (34–173)	126 (46–212)	0.16
CRP, mg/l	24 (6–63)	67 (15–123)	<0.01
BVAS	14 (10–18)	15 (12–19)	0.33
Kidney biopsy	(*n* = 38)	(*n* = 37)	
Berden class			
Focal	11 (29)	11 (30)	0.31
Crescentic	7 (18)	11 (30)	
Mixed	15 (40)	14 (38)	
Sclerotic	5 (13)	1 (2)	
AKRiS			
Low	21 (55)	19 (51)	0.44
Moderate	15 (40)	12 (32)	
High	2 (5)	5 (14)	
Very high	0	1 (3)	
Treatment			
Induction therapy			
Cyclophosphamide, g	1.4 (1.0–2.0)	1.0 (1.0–2.0)	0.86
Rituximab, g	2.0 (2.0–2.0)	2.0 (2.0–2.0)	0.23
Plasma exchange, *n* (%)	1 (2)	7 (13)	0.07
Prednisolone			
Cumulative dose, g	1.1 (0.9–1.6)	2.9 (2.2–3.3)	<0.001
Duration, wks	8.0 (6.0–9.0)	39.0 (28.0–52.5)	<0.001
Outcomes			
Remission, n (%)	51 (91)	56 (100)	0.07
Time to remission, d	71 (57–85)	81 (70–101)	<0.01
Relapse, *n* (%)	0	5 (9)	0.09
New kidney failure	0	0	1.00
Death	8 (14)	9 (16)	1.00
Adverse events			
Weight gain^a^	6 (23)	7 (32)	0.72
Hypogammaglobulinemia^a^			
Moderate	1 (2)	4 (10)	0.20
Severe	1 (2)	0	
Lymphopenia^a^	12 (24)	22 (39)	0.14
Neutropenia^a^	2 (4)	3 (5)	1.00
COVID-19	6 (11)	3 (5)	0.49
Infections^b^			
Number of patients	4 (7)	12 (21)	0.06
Number of events	12 (21)	21 (38)	0.09
Malignancy	0	4 (7)	0.13
Cardiovascular disease	2 (3)	6 (11)	0.27
Osteoporosis	2 (4)	1 (2)	1.00
Cataracts	1 (2)	4 (7)	0.36

AKRiS, ANCA Kidney Risk Score; ANCA, antineutrophil cytoplasmic antibody; BVAS, Birmingham Vasculitis Activity Score; CRP, C-reactive protein; eGFR, estimated glomerular filtration rate; EGPA, eosinophilic GPA; GPA, granulomatosis with polyangiitis; MPA, microscopic polyangiitis; MPO, myeloperoxidase; PR3, proteinase 3; uPCR, urinary protein-to-creatinine ratio.

Data are presented as mean ± SD, median (interquartile range), or number of patients (%).

Missing data: ≤ 12 wks subgroup: baseline weight = 18; CRP = 2, uPCR = 3, weight gain = 30, hypogammaglobulinemia = 11, lymphopenia = 6, neutropenia = 6. > 12 wks subgroup: baseline weight = 26; uPCR = 9; weight gain = 34; hypogammaglobulinemia = 17.

a At 6 months.

b Non-COVID-19 infection requiring hospitalization.

There was no difference in the number of patients achieving clinical remission between the 2 groups (glucocorticoids for > 12 weeks vs. ≤ 12 weeks: 56/56 [100%] vs. 51/56 [91%], *P* = 0.07). However, the time to disease remission was shorter in patients receiving ≤ 12 weeks of glucocorticoid therapy (*P* = 0.04; Figure 1b). The 5 patients who did not achieve disease remission died within 4 months of diagnosis and the causes for mortality were not attributable to underprescribing of glucocorticoids (Supplementary Table S3). Disease relapse rate was similar between the 2 groups (glucocorticoids for > 12 weeks vs. ≤ 12 weeks: 5/56 [9%] vs. 0/56 [0%], *P* = 0.07). Finally, there was no difference in renal recovery (specifically, improvement in eGFR) between those treated with glucocorticoids for > 12 weeks compared with those treated with glucocorticoids for ≤ 12 weeks (*P* = 0.48) (Figure 2a).

### Subgroup Analyses

We performed several subgroup analyses to further characterize the cohort and response to treatment. These included evaluating our findings only in those patients with a new presentation of AAV (i.e., excluding relapsing disease) and only in those with a new presentation of AAV affecting the kidney. Overall, the findings were consistent with the overall cohort (Supplementary Tables S4–S7).

To determine whether disease severity was associated with oral glucocorticoid dose or duration, we stratified these in patients with kidney involvement according to baseline eGFR, degree of proteinuria, and initial kidney biopsy disease classification. No association between any of these severity markers and oral glucocorticoid dose or duration were observed (Supplementary Figure S4).

### Adverse Events

Adverse events are shown in Table 2. There was no significant hypogammaglobulinemia despite rituximab induction and maintenance therapy. Mild lymphopenia persisted throughout the follow-up period (Supplementary Figure S3). Twenty-two patients (20%) developed infection requiring hospitalization, of whom 6 (27%) had isolated COVID-19 infection. The etiology of all infections is shown in Supplementary Table S8. Four malignancies were diagnosed during the follow-up period as follows: nonmelanoma skin cancer, bladder transitional cell carcinoma, ovarian cancer, and pancreatic cancer.

There were no significant differences in adverse events and mortality between patients receiving > or ≤ 12 weeks of glucocorticoid therapy. There was a trend toward a lower rate of non-COVID-19 infectious complications in those treated with shorter duration glucocorticoids (7% of patients in those receiving ≤ 12 weeks glucocorticoid therapy vs. 21% in those receiving > 12 weeks; *P* = 0.06).

## Discussion

Despite advances in the management of AAV, there remain several key clinical challenges. Among these is the toxicity of long-term immunosuppression, particularly glucocorticoids. Although widely used in the management of AAV, prolonged glucocorticoid use (including the use of i.v. methylprednisolone) is associated with the development of complications, including cardiovascular disease, diabetes mellitus, infections, and osteoporosis.^5^, ^6^, ^7^^,^^10^ Here, our real-world findings in a large cohort of patients with AAV demonstrate that induction treatment with combination cyclophosphamide-rituximab, followed by rituximab maintenance, permits early glucocorticoid withdrawal and provides excellent disease control, a low risk of disease relapse, and an acceptable safety profile, without the need for i.v. glucocorticoids.

Recent randomized controlled trials have evaluated reduced-dose glucocorticoid regimens in severe AAV. The PEXIVAS trial randomized > 700 patients with moderate to severe kidney impairment (eGFR < 50 ml/min per 1.73 m^2^) to a standard versus reduced-dose glucocorticoid taper (cumulative dose over first 12 months ∼4.9 g vs. ∼3.2 g oral glucocorticoid) following induction therapy with i.v. methylprednisolone (1–3 g) and either cyclophosphamide or rituximab.^9^ Over a median of 2.9 years follow-up, the reduced-dose regimen was noninferior for the primary outcome of kidney failure or death, with fewer serious infections. Most recently, the ADVOCATE trial investigated complement blockade as a substitute for glucocorticoid treatment, using the novel C5aR1 antagonist, avacopan.^24^ The avacopan-based regimen was noninferior to glucocorticoids for the induction of disease remission at 6 months and superior for sustained remission at 12 months, and was associated with fewer adverse events. However, the avacopan group still received a cumulative glucocorticoid dose of 1.7 g, including the use of i.v. methylprednisolone. Finally, the LOVAS study demonstrated efficacy of a low-dose (0.5 mg/kg; total dose: 1.3 g) glucocorticoid induction regimen alongside rituximab monotherapy; however, these patients had mild disease (median eGFR: 52–55 ml/min per 1.73 m^2^), were predominantly myeloperoxidase-ANCA positive (85%), and 1 in 4 patients continued to have active disease at 6 months.^25^

Although these studies demonstrate that glucocorticoid doses may be successfully tapered during remission-induction, overall glucocorticoid burden remained significant. This is important when considering recent uncontrolled studies that suggest that a very rapid glucocorticoid taper may be feasible without the use of avacopan or other adjunctive therapies, and instead using an induction regimen combining cyclophosphamide and rituximab.^15^^,^^26^ This combination was originally tested in the RITUXVAS study where it was compared with a standard cyclophosphamide regimen and “standard” glucocorticoid taper.^27^ RITUXVAS found that the rituximab-based regimen, which included at least 2 doses of cyclophosphamide, was noninferior to standard therapy for inducing remission in severe AAV. In our study, we show that a similar combination of rituximab and 2 doses of cyclophosphamide, but with no i.v. glucocorticoid and early oral glucocorticoid withdrawal, provides robust disease control. Interestingly, our cohort experienced a lower rate of adverse events than in RITUXVAS, suggesting the adverse events in the trial may have been driven by the glucocorticoid therapy or by patients in RITUXVAS having more severe disease (9%–20% dialysis-dependent at randomization) as these patients are recognized to have greatest infection risk.

Alongside RITUXVAS, McAdoo *et al.*^26^ have shown that a similar approach (i.v. cyclophosphamide [3 g], rituximab [2 g], and a 1–2-week course of glucocorticoid [total dose: ∼1.2 g]) may provide rapid and prolonged disease control without increasing infection risk.^15^^,^^26^ Our findings, which are in a larger group of patients without exclusive kidney disease, are in line with these earlier findings. In a representative group of patients with AAV (median age: 67 years; ∼50% microscopic polyangiitis; 85% kidney involvement with median eGFR of 24 ml/min per 1.73 m^2^), low-dose glucocorticoid (1.7 g) achieved a similar, if not superior, disease remission rate (95%) to that seen in these studies. Importantly, in comparison to the earlier studies which used azathioprine as maintenance immunosuppression, our use of rituximab is more in line with current practice. In addition, our group received no i.v. glucocorticoid therapy compared with 82% of patients in the study by Pepper *et al.*^15^

No controlled studies have defined the optimal duration of maintenance glucocorticoids in AAV. Current data suggest an increased risk of disease relapse following glucocorticoid withdrawal.^11^^,^^28^ However, these findings predate the widespread use of rituximab for remission-maintenance. Importantly, in studies investigating rituximab as a maintenance therapy, many patients continue to receive oral glucocorticoid, and its contribution to long-term disease control in this setting warrants further investigation. The Assessment of Prednisolone In Remission study will be informative in this regard; it is prospectively evaluating glucocorticoid withdrawal in patients with GPA, including those receiving rituximab maintenance.^29^ It has completed recruitment, and its results are eagerly awaited.

Previous data suggest that patients with kidney failure, including those on dialysis at disease presentation, have poor long-term outcomes. For example, in the MEPEX study, 1 in 3 patients who were on dialysis at the time of recruitment remained dialysis-dependent at 12 months.^30^ Our data show excellent kidney outcomes at 1 year with a strong negative correlation demonstrated between baseline eGFR and improvement in eGFR at 12 months. This supports treatment for patients with kidney failure at disease presentation and emphasizes the role of kidney biopsy in combination with serum creatinine in disease prognostication.

Interestingly, we found no difference in disease outcomes between those who received > or < 12 weeks of glucocorticoids despite a significant difference in cumulative dose (2.8 g vs. 1.1 g). We recognize that glucocorticoid tapering and withdrawal were not protocolized in our study and that, despite similar baseline characteristics, there may be unadjusted confounders between these groups. However, our data suggest that some patients may be able to stop glucocorticoids earlier than the guidelines currently recommend.^2^ As expected, our data demonstrate patients with eosinophilic GPA require longer duration glucocorticoid therapy. Importantly, our findings suggest that initial treatment with i.v. methylprednisolone may be unnecessary in the early management of AAV. This not only increases cumulative glucocorticoid exposure but is also associated with an increased risk of infection and diabetes.^10^ Our data add to clinical equipoise over both the need for i.v. glucocorticoids and the optimal duration of oral glucocorticoid therapy in patients with severe AAV, both of which should be robustly examined in a randomized clinical trial.

Our study has limitations inherent to its retrospective design, including potential selection bias and the inability to control for all confounding variables. In addition, the generalizability of our findings may be limited by the single-center nature of our cohort. Nonetheless, the long-term follow-up of approximately 3 years provides valuable insights into the durability of remission and the safety of glucocorticoid minimization strategies in AAV. Future randomized controlled trials are warranted to confirm these findings and explore even lower glucocorticoid doses or alternative immunosuppressive combinations that may further mitigate treatment-related morbidity.

## Disclosure

All the authors declared no competing interests.

## Data Availability Statement

The data that support the findings of this study are available from the corresponding author [ND] upon request and subject to satisfactory data-sharing agreements that preserve patient confidentiality.

## Supplementary Material

Supplementary File (PDF) **Figure S1.** Study population. **Figure S2.** Edinburgh Vasculitis Service Treatment Protocol. **Figure S3.** Trends in immunological and biochemical markers following treatment of AAV. **Figure S4.** Oral glucocorticoid therapy and markers of disease severity. **Table S1.** Trimethoprim/sulphamethoxazole desensitization protocol. **Table S2.** Baseline characteristics of patients based on ANCA serotype. **Table S3.** Causes of early mortality with active AAV. **Table S4.** Baseline characteristics of whole cohort versus new presentations of AAV only. **Table S5.** Patients with new presentation of AAV stratified by oral glucocorticoid duration. **Table S6.** Baseline characteristics of all patients with new presentations of AAV versus only those with kidney involvement. **Table S7.** Patients with new presentation of AAV involving the kidney stratified by oral glucocorticoid duration. **Table S8.** Etiology of infectious complications. STROBE checklist.

## Footnotes

Supplementary File (PDF)
**Figure S1.** Study population. **Figure S2.** Edinburgh Vasculitis Service Treatment Protocol. **Figure S3.** Trends in immunological and biochemical markers following treatment of AAV. **Figure S4.** Oral glucocorticoid therapy and markers of disease severity. **Table S1.** Trimethoprim/sulphamethoxazole desensitization protocol. **Table S2.** Baseline characteristics of patients based on ANCA serotype. **Table S3.** Causes of early mortality with active AAV. **Table S4.** Baseline characteristics of whole cohort versus new presentations of AAV only. **Table S5.** Patients with new presentation of AAV stratified by oral glucocorticoid duration. **Table S6.** Baseline characteristics of all patients with new presentations of AAV versus only those with kidney involvement. **Table S7.** Patients with new presentation of AAV involving the kidney stratified by oral glucocorticoid duration. **Table S8.** Etiology of infectious complications. STROBE checklist.
