# Supplementary material for: Combination Cyclophosphamide and Rituximab to Minimize Glucocorticoid Use in Antineutrophil Cytoplasm Antibody–Associated Vasculitis
Source: Kidney Int Rep. 2025 Apr 22;10(7):2334–43. doi: 10.1016/j.ekir.2025.04.030 (PMC12266221; doi:10.1016/j.ekir.2025.04.030)
Supplement: Supplementary File (PDF) — Figure S1. Study population. Figure S2. Edinburgh Vasculitis Service Treatment Protocol. Figure S3. Trends in immunological and biochemical markers following treatment of AAV. Figure S4. Oral glucocorticoid therapy and markers of disease severity. Table S1. Trimethoprim/sulphamethoxazole desensitization protocol. Table S2. Baseline characteristics of patients based on ANCA serotype. Table S3. Causes of early mortality with active AAV. Table S4. Baseline characteristics of whole cohort versus new presentations of AAV only. Table S5. Patients with new presentation of AAV stratified by oral glucocorticoid duration. Table S6. Baseline characteristics of all patients with new presentations of AAV versus only those with kidney involvement. Table S7. Patients with new presentation of AAV involving the kidney stratified by oral glucocorticoid duration. Table S8. Etiology of infectious complications. STROBE checklist. [file mmc1.pdf]

Supplementary Material

Supplementary Tables

Supplementary table S1. ***Trimethoprim/sulphamethoxazole desensitisation protocol.***

**Step**	**Dose (480 mg /5 ml)**
Days 1-3	80 mg sulfamethoxazole + 16 mg trimethoprim (1 ml oral suspension)
Days 4-6	160 mg sulfamethoxazole + 32 mg trimethoprim (2 ml oral suspension)
Days 7-9	240 mg sulfamethoxazole + 48 mg trimethoprim (3 ml oral suspension)
Days 10-12	320 mg sulfamethoxazole + 64 mg trimethoprim (4 ml oral suspension)
Day 13 onwards	400 mg sulfamethoxazole + 80 mg trimethoprim (1x 480 mg tablet)

Supplementary table S2. ***Baseline characteristics of patients based on ANCA serotype.***

**Characteristics**		**PR3 positive (n=46)**	**MPO positive (n=47)**	**ANCA negative (n=14)**	**Dual positive (n=5)**
Age – years		64 (49-72)	71 (63-78)	63 (54-76)	74 (64-78)
Male sex		30 (65)	25 (53)	7 (50)	2 (40)
Organ involvement	ENT	15 (33)	6 (13)	2 (14)	0
	Respiratory	32 (70)	28 (60)	6 (43)	2 (40)
	Pulmonary haemorrhage	7 (15)	4 (9)	3 (21)	1 (20)
	Renal	41 (89)	40 (85)	10 (71)	4 (80)
	eGFR <15 mL/min/1.73m^2 or requiring dialysis	7 (15)	9 (19)	1 (7)	0
	Nervous system	11 (24)	7 (15)	6 (43)	1 (20)
	Ocular	10 (22)	1 (2)	1 (7)	1 (20)
	Cutaneous	8 (17)	0	1 (7)	0
	Rheumatologic	11 (24)	3 (6)	1 (7)	0
AAV phenotype	GPA	41 (89)	2 (4)	4 (29)	1 (20)
	MPA	2 (4)	43 (92)	9 (64)	4 (80)
	EGPA	3 (7)	2 (4)	1 (7)	0
Baseline biochemistry	Creatinine - μmol/L	170 (100-332)	222 (110-398)	109 (65-227)	161 (132-197)
	eGFR – mL/min/1.73^2	38 (13-74)	24 (13-50)	69 (20-91)	44 (24-46)
	uPCR – mg/mmol	101 (60-198)	103 (39-175)	143 (16-283)	34 (17-46)
	CRP – mg/L	65 (10-125)	46 (5-76)	17 (8-50)	34 (17-38)
Disease activity (BVAS)		14.5 (12.0-21.0)	16.0 (11.0-19.0)	11.5 (8.3-16.5)	13.0 (11.0-15.0)
**Kidney biopsy**		35 (76)	33 (70)	5 (36)	2 (40)
Berden class	Focal	12 (34)	6 (18)	2 (40)	2 (100)
	Crescentic	11 (31)	7 (21)	0	0
	Mixed	10 (29)	16 (49)	3 (60)	0
	Sclerotic	2 (6)	4 (12)	0	0
AKRiS	Low	19 (54)	14 (42)	5 (100)	2 (100)
	Moderate	14 (40)	13 (39)	0	0
	High	2 (6)	5 (16)	0	0
	Very High	0	1 (3)	0	0
**Treatment**					
Induction therapy	Cyclophosphamide – g	2.0 (1.0-2.0)	1.0 (1.0-2.0)	1.2 (1.0-2.0)	1.0 (1.0-2.0)
	Rituximab – g	2.0 (2.0-2.0)	2.0 (2.0-2.0)	2.0 (2.0-2.0)	2.0 (2.0-2.0)
	Plasma exchange	5 (10.9)	2 (4.3)	1 (7.1)	0

Oral prednisolone	Cumulative dose – g<br>Duration – weeks	1.9 (1.2-3.0)<br>13.5 (8.0-41.8)	1.7 (1.1-3.0)<br>10.0 (7.5-41.5)	2.6 (1.8-2.9)<br>27.5 (19.3-38.5)	1.0 (0.8-2.1)<br>9.0 (7.0-28.0)
**Outcomes**					
Remission		43 (94)	45 (96)	13 (93)	5 (100)
Time to remission – days		78 (57-98)	74 (59-85)	81 (75-88)	84 (70-91)
Relapse		3 (6)	1 (2)	1 (7)	0

Data are presented as mean±SD, median (interquartile range), or number of patients (%).
AAV: ANCA-associated vasculitis; AKRiS: ANCA Kidney Risk Score; ANCA: Anti-neutrophil cytoplasmic antibody; BVAS: Birmingham Vasculitis Activity Score; CRP: C-reactive protein; eGFR: estimated glomerular filtration rate; EGPA: eosinophilic granulomatosis with polyangiitis; ENT: Ear, nose and throat; GPA: granulomatosis with polyangiitis; MPA: microscopic polyangiitis; MPO: myeloperoxidase; PR3: proteinase 3; uPCR: urinary protein:creatinine

Supplementary table S3. ***Causes of early mortality with active AAV.***

**Case number**	**Cause of mortality**	**Additional details**
1	COVID-19 pneumonitis	None
2	Pneumonia and empyema	None
3	Diverticular perforation and peritonitis	None
4	Advanced renal failure at presentation	Unsuitable for RRT (cardiac and respiratory co-morbidities)
5	Advanced renal failure at presentation	Unsuitable for RRT (cardiac and respiratory co-morbidities)

Supplementary table S4. ***Baseline characteristics of whole cohort versus new presentations of AAV only.***

**Characteristics**		**Whole cohort (n=112)**	**New diagnosis only (n=98)**
Age – years		67 (56-76)	67 (57-75)
Male sex – no. (%)		64 (57)	56 (57)
Disease status	First presentation	98 (88)	98 (100)
	Relapsed disease	14 (13)	0
Organ involvement	ENT	23 (21)	21 (21)
	Respiratory	68 (61)	59 (60)
	Pulmonary haemorrhage	15 (13)	9 (9)
	Renal	95 (85)	84 (86)
	eGFR <15 mL/min/1.73m^2 or requiring dialysis	34 (30)	32 (33)
	Nervous system	25 (22)	21 (21)
	Ocular	13 (12)	10 (10)
	Cutaneous	9 (8)	7 (7)
	Rheumatologic	15 (13)	13 (13)
AAV phenotype	GPA	48 (43)	41 (42)
	MPA	58 (52)	51 (52)
	EGPA	6 (5)	6 (6)
ANCA serology	PR3	46 (41)	39 (40)
	MPO	47 (42)	44 (45)
	Dual PR3/MPO positive	5 (5)	3 (3)
	Negative	14 (13)	12 (12)
Baseline biochemistry	Creatinine – μmol/L	183 (98-349)	216 (105-358)
	eGFR – mL/min/1.73m^2	28 (14-70)	26 (13-63)
	uPCR – mg/mmol	101 (36-198)	108 (40-198)
	CRP – mg/L	44 (8-101)	48 (8-103)
Disease activity (BVAS)		15 (11-19)	15 (11-19)
**Kidney biopsy**		(n=75)	(n=71)
Berden class	Focal	22 (29)	21 (30)
	Crescentic	18 (24)	18 (25)
	Mixed	29 (39)	27 (38)
	Sclerotic	6 (8)	5 (7)
AKRiS	Low	40 (53)	37 (52)
	Moderate	27 (36)	26 (37)
	High	7 (9)	7 (10)
	Very high	1 (1)	1 (1)
**Treatment**			
Induction therapy	Cyclophosphamide – g	1.0 (1.0-2.0)	1.2 (1.0-2.0)
	Rituximab – g	2.0 (2.0-2.0)	2.0 (2.0-2.0)
	Plasma exchange - n (%)	8 (7)	8 (8)
Maintenance agent	Rituximab	100 (89)	86 (88)
	Azathioprine	2 (2)	2 (2)
	None	10 (9)	10 (10)
Oral prednisolone	Cumulative dose – g	1.8 (1.1-2.9)	1.8 (1.2-2.9)
	Duration – weeks	12.5 (8.0-39.0)	12.0 (8.0-39.5)

Data are presented as median (interquartile range) or number of patients (%).
Missing data: whole cohort: uPCR=12; CRP=2. New diagnosis only: uPCR=10; CRP=2.

AAV: ANCA-associated vasculitis; AKRiS: ANCA Kidney Risk Score; ANCA: Anti-neutrophil cytoplasm antibody; BVAS: Birmingham Vasculitis Activity Score; CRP: C-reactive protein; eGFR: estimated glomerular filtration rate; EGPA: eosinophilic granulomatosis with polyangiitis; ENT: Ear, nose and throat; GPA: granulomatosis with polyangiitis; MPA: microscopic polyangiitis; MPO: myeloperoxidase; PR3: proteinase 3; uPCR: urinary protein:creatinine ratio.

Supplementary table S5. ***Patients with new presentation of AAV stratified by oral glucocorticoid duration.***

**Characteristics**		**≤12 weeks (N=50)**	**>12 weeks (N=48)**	**P-value**
Age – years		70 (62-77)	65 (51-74)	0.09
Male sex – no. (%)		27 (54)	29 (60)	0.66
Baseline weight – kg		71.6±17.4	71.1±15.0	0.91
Organ involvement	ENT	8 (16)	13 (27)	0.28
	Respiratory	27 (54)	32 (67)	0.28
	Pulmonary haemorrhage	3 (6)	6 (13)	0.45
	Renal	42 (84)	42 (88)	0.84
	eGFR <15 mL/min/1.73m² or requiring dialysis	17 (34)	15 (31)	0.94
	Nervous system	10 (20)	11 (23)	0.92
	Ocular	4 (8)	6 (13)	0.69
	Cutaneous	4 (8)	3 (6)	1.0
	Rheumatologic	7 (14)	6 (13)	1.0
ANCA serology	PR3	18 (36)	21 (44)	0.01
	MPO	27 (54)	17 (35)	
	Dual positive	3 (6)	0	
	Negative	2 (4)	10 (21)	
AAV phenotype	GPA	20 (40)	21 (44)	0.02
	MPA	30 (60)	21 (44)	
	EGPA	0	6 (13)	
Baseline biochemistry	Creatinine – μmol/L	197 (111-347)	226 (102-380)	0.76
	eGFR – mL/min/1.73m²	27 (14-54)	26 (13-72)	0.96
	uPCR – mg/mmol	86 (34-160)	143 (75-217)	0.04
	CRP – mg/L	29 (7-62)	86 (19-129)	<0.01
BVAS		14 (10-18)	17 (12-20)	0.20
**Kidney biopsy**		(n=35)	(n=36)	
Berden class	Focal	10 (29)	11 (31)	0.55
	Crescentic	7 (20)	11 (31)	
	Mixed	14 (40)	13 (36)	
	Sclerotic	4 (11)	1 (2)	
AKRiS	Low	18 (51)	19 (53)	0.52
	Moderate	15 (43)	11 (31)	
	High	2 (6)	5 (14)	
	Very high	0	1 (3)	
**Treatment**				
Induction therapy	Cyclophosphamide – g	1.4 (1.0-2.0)	1.0 (1.0-2.0)	0.90
	Rituximab – g	2.0 (2.0-2.0)	2.0 (2.0-2.0)	0.24
	Plasma exchange – n (%)	1 (2.0)	7 (14.6)	0.06
Prednisolone	Cumulative dose – g	1.2 (0.9-1.6)	2.9 (2.2-3.4)	<0.001
	Duration – weeks	8.0 (6.0-9.0)	41.5 (28.0-52.5)	<0.001
**Outcomes**				
Remission – no. (%)		45 (90.0)	48 (100)	0.07
Time to remission –days		75 (65-85)	81 (70-99)	0.07

Relapse – no. (%)		0	5 (10)	0.08
New kidney failure*		0	0	1.00
Death		8 (16)	8 (17)	1.00
**Adverse events**				
Weight gain*		5 (24)	7 (44)	0.35
Hypogammaglobulinemia*				0.18
	Moderate	1 (3)	4 (13)	
	Severe	1 (3)	0	
Lymphopenia*		12 (27)	19 (40)	0.27
Neutropenia*		2 (4)	3 (6)	1.00
COVID-19		6 (12)	3 (6)	0.49
Infections*				
	Number of patients	4 (8)	10 (21)	0.09
	Number of events	6 (12)	15 (31)	0.02
Malignancy		0	4 (8)	0.12
Cardiovascular disease		2 (4)	5 (10)	0.40
Osteoporosis		2 (4)	1 (2)	0.97
Cataracts		1 (2)	4 (8)	0.33

Data are presented as mean±SD, median (interquartile range), or number of patients (%).
*at 6 months; **non-COVID-19 infection requiring hospitalisation.
Missing data: ≤12 weeks subgroup: baseline weight=18; CRP=2; uPCR=3; weight gain=29; hypogammaglobulinaemia=10; lymphopenia=5; neutropenia=5. >12 weeks subgroup: baseline weight=25; uPCR=7; weight gain=32; hypogammaglobulinaemia=16.
AKRiS: ANCA Kidney Risk Score; ANCA: Antineutrophil Cytoplasmic Antibody; BVAS: Birmingham Vasculitis Activity Score; CRP: C-reactive protein; COVID-19: Coronavirus disease 2019; eGFR: estimated glomerular filtration rate; EGPA: eosinophilic granulomatosis with polyangiitis, GPA: granulomatosis with polyangiitis, MPA: microscopic polyangiitis; MPO: myeloperoxidase; PR3: proteinase 3; uPCR: urinary protein:creatinine ratio

Supplementary table S6. ***Baseline characteristics of all patients with new presentations of AAV versus only those with kidney involvement.***

Characteristics		New diagnosis (n=98)	Kidney only (n=84)
Age – years		67 (57-75)	67 (54-75)
Male sex – no. (%)		56 (57)	52 (62)
Disease status	First presentation	98 (100)	84 (100)
	Relapsed disease		
Organ involvement	ENT	21 (21)	18 (21)
	Respiratory	59 (60)	51 (61)
	Pulmonary haemorrhage	9 (9)	9 (11)
	Renal	84 (86)	84 (100)
	eGFR <15 mL/min/1.73m^2 or requiring dialysis	32 (33)	32 (38)
	Nervous system	21 (21)	17 (20)
	Ocular	10 (10)	9 (11)
	Cutaneous	7 (7)	7 (8)
	Rheumatologic	13 (13)	11 (13)
AAV phenotype	GPA	41 (42)	35 (42)
	MPA	51 (52)	45 (54)
	EGPA	6 (6)	4 (5)
ANCA serology	PR3	39 (40)	36 (43)
	MPO	44 (45)	38 (45)
	Dual PR3/MPO positive	3 (3)	2 (2)
	Negative	12 (12)	8 (10)
Baseline biochemistry	Creatinine – μmol/L	216 (105-358)	235 (136-389)
	eGFR – mL/min/1.73m^2	26 (13-63)	23 (13-44)
	uPCR – mg/mmol	108 (40-198)	119 (61-210)
	CRP – mg/L	48 (8-103)	55 (13-105)
Disease activity (BVAS)		15 (11-19)	17 (12-20)
**Kidney biopsy**		(n=71)	(n=71)
Berden class	Focal	21 (30)	21 (30)
	Crescentic	18 (25)	18 (25)
	Mixed	27 (38)	27 (38)
	Sclerotic	5 (7)	5 (7)
AKRiS	Low	37 (52)	37 (52)
	Moderate	26 (37)	26 (37)
	High	7 (10)	7 (10)
	Very high	1 (1)	1 (1)
**Treatment**			
Induction therapy	Cyclophosphamide – g	1.2 (1.0-2.0)	1.0 (1.0-2.0)
	Rituximab – g	2.0 (2.0-2.0)	2.0 (2.0-2.0)
	Plasma exchange – n (%)	8 (8)	8 (10)
Maintenance agent	Rituximab	86 (88)	72 (86)
	Azathioprine	2 (2)	2 (2)
	None	10 (10)	10 (12)
Oral prednisolone	Cumulative dose – g	1.8 (1.1-2.9)	1.8 (1.1-2.9)
	Duration – weeks	12.0 (8.0-39.5)	12.0 (7.8-40.1)

Data are presented as median (interquartile range) or number of patients (%).
Missing data: New diagnosis: uPCR=10; CRP=2. Kidney only: uPCR=6; CRP=1.

AAV: ANCA-associated vasculitis; AKRiS: ANCA Kidney Risk Score; ANCA: Anti-neutrophil cytoplasm antibody; BVAS: Birmingham Vasculitis Activity Score; CRP: C-reactive protein; eGFR: estimated glomerular filtration rate; EGPA: eosinophilic granulomatosis with polyangiitis; ENT: Ear, nose and throat; GPA: granulomatosis with polyangiitis; MPA: microscopic polyangiitis; MPO: myeloperoxidase; PR3: proteinase 3; uPCR: urinary protein:creatinine ratio.

Supplementary table S7. ***Patients with new presentation of AAV involving the kidney stratified by oral glucocorticoid duration.***

**Characteristics**		**≤12 weeks (n=42)**	**>12 weeks (n=42)**	**P-value**
Age – years		71 (63-77)	63 (50-72)	0.02
Male sex – no. (%)		24 (57)	28 (66.7)	0.50
Baseline weight – kg		71.5±17.6	74.1±13.5	0.59
Organ involvement	ENT	6 (14)	12 (28.6)	0.18
	Respiratory	23 (55)	28 (66.7)	0.37
	Pulmonary haemorrhage	3 (7)	6 (14.3)	0.48
	Renal	42 (100)	42 (100)	
	eGFR <15 mL/min/1.73m^2 or requiring dialysis	9 (21)	7 (16.7)	0.78
	Nervous system	8 (19)	9 (21.4)	1.00
	Ocular	4 (10)	5 (11.9)	1.00
	Cutaneous	4 (10)	3 (7.1)	1.00
	Rheumatologic	5 (12)	6 (14.3)	1.00
ANCA serology	PR3	16 (38)	20 (47.6)	0.04
	MPO	23 (55)	15 (35.7)	
	Dual positive	2 (5)	0	
	Negative	1 (2)	7 (16.7)	
AAV phenotype	GPA	16 (38)	19 (45.2)	0.07
	MPA	26 (62)	19 (45.2)	
	EGPA	0	4 (9.6)	
Baseline biochemistry	Creatinine – μmol/L	225 (153-362)	248 (124-416)	0.72
	eGFR – mL/min/1.73m^2	23 (13-42)	23 (12-52)	0.96
	uPCR – mg/mmol	100 (43-174)	163 (100-236)	0.03
	CRP – mg/L	34 (7-63)	90 (38-126)	<0.01
BVAS		16 (11-19)	18 (13-21)	0.18
**Kidney biopsy**		(n=35)	(n=36)	
Berden class	Focal	10 (29)	11 (30.6)	0.58
	Crescentic	7 (20)	11 (30.6)	
	Mixed	14 (40)	13 (36.1)	
	Sclerotic	4 (11)	1 (2.7)	
AKRiS	Low	18 (51)	19 (52.8)	0.56
	Moderate	15 (43)	11 (30.6)	
	High	2 (6)	5 (13.9)	
	Very high	0	1 (2.7)	
**Treatment**				
Induction therapy	Cyclophosphamide – g	1.4 (0.8-2.0)	1.0 (1.0-2.0)	0.82
	Rituximab – g	2.0 (2.0-2.0)	2.0 (2.0-2.0)	0.21
	Plasma exchange – n (%)	1 (2)	7 (16.7)	0.06
Prednisolone	Cumulative dose – g	1.1 (0.8-1.6)	2.9 (2.4-3.4)	<0.001
	Duration – weeks	7.5 (6.0-8.8)	41.5 (28.0-54.0)	<0.001
**Outcomes**				
Remission – no. (%)		37 (88)	42 (100)	0.07
Time to remission –days		77 (59-90)	81 (69-100)	0.13
Relapse – no. (%)		0	4 (10)	0.17

New kidney failure*		0	0	1.00
Death		8 (19)	8 (19)	1.00
**Adverse events**				
Weight gain*		5 (28)	7 (47)	0.45
Hypogammaglobulinemia*				0.50
	Moderate	1 (3)	2 (8)	
	Severe	1 (3)	0	
Lymphopenia*		11 (30)	16 (38)	0.59
Neutropenia*		2 (5)	3 (7)	1.00
COVID-19		6 (14)	3 (7)	0.48
Infections**				
	Number of patients	4 (10)	10 (24)	0.14
	Number of events	6 (14)	15 (36)	0.04
Malignancy		0	4 (10)	0.12
Cardiovascular disease		2 (5)	4 (10)	0.42
Osteoporosis		2 (5)	1 (2)	1.00
Cataracts		1 (2)	3 (7)	0.62

Data are presented as mean±SD, median (interquartile range), or number of patients (%).

*at 6 months; **non-COVID-19 infection requiring hospitalisation.

Missing data: ≤12 weeks subgroup: baseline weight=13; CRP=1; uPCR=1; weight gain=24; hypogammaglobulinaemia=10; lymphopenia=5; neutropenia=5. >12 weeks subgroup: baseline weight=23; uPCR=5; weight gain=27; hypogammaglobulinaemia=16.

AKRiS: ANCA Kidney Risk Score; ANCA: Antineutrophil Cytoplasmic Antibody; BVAS: Birmingham Vasculitis Activity Score; CRP: C-reactive protein; COVID-19: Coronavirus disease 2019; eGFR: estimated glomerular filtration rate; EGPA: eosinophilic granulomatosis with polyangiitis, GPA: granulomatosis with polyangiitis, MPA: microscopic polyangiitis; MPO: myeloperoxidase; PR3: proteinase 3; uPCR: urinary protein:creatinine ratio

Supplementary table S8. ***Aetiology of infectious complications.***

**Aetiology of infection**	**Events**
Pneumonia	12 (11)
Viral infections	
COVID-19	9 (8)
Other respiratory viruses	2 (2)
Herpes Zoster virus	1 (1)
CMV	1 (1)
UTI	3 (3)
Cellulitis	2 (2)
Peritoneal dialysis peritonitis	1 (1)
Periodontal infection	1 (1)
Unknown source	1 (1)

Data are presented as number of events (%).

Supplementary Figures

Supplementary figure S1. ***Study population.***

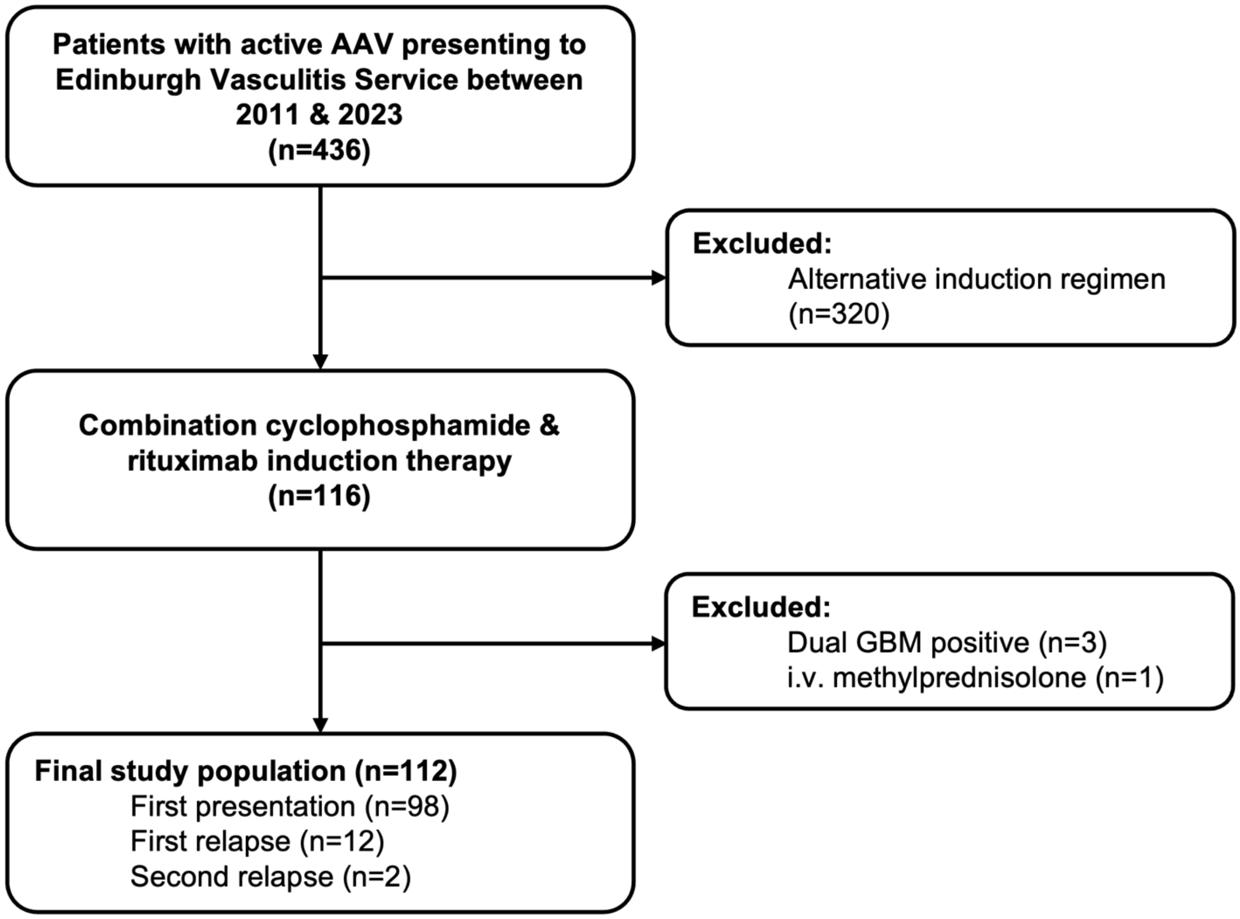

Supplementary figure S2. ***Edinburgh Vasculitis Service Treatment Protocol.*** *Mesna dose is 40% of the cyclophosphamide dose and is given in the same bag as the cyclophosphamide. Cyclophosphamide dose is age- and eGFR adjusted (usual dose per infusion 0.5-1 g). eGFR: estimated glomerular filtration rate; i.v.: intravenous; PPI: proton pump inhibitor.

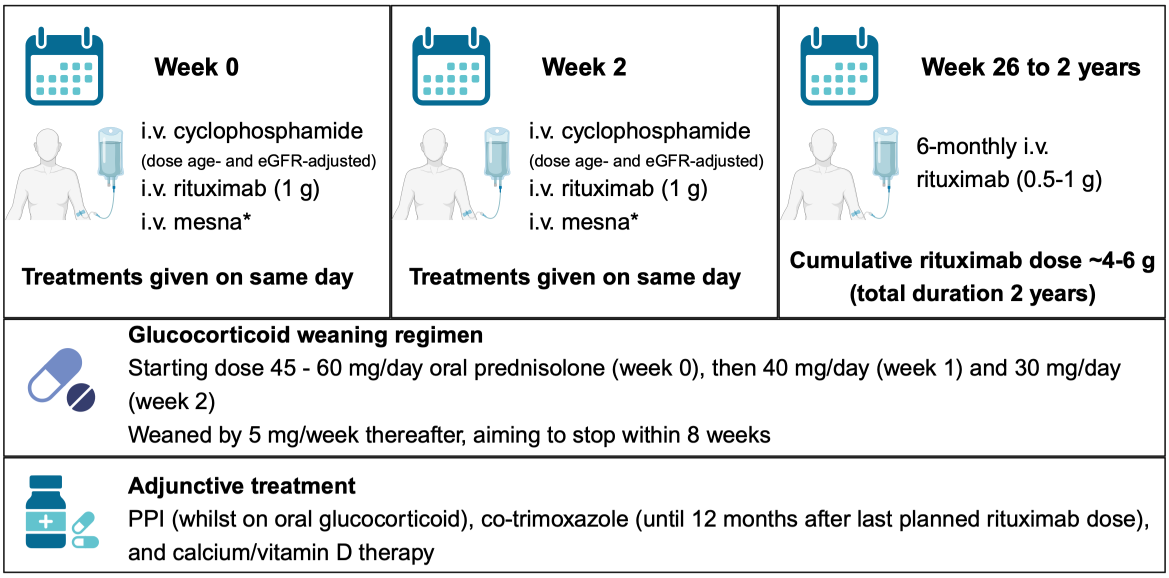

Supplementary figure S3. ***Trends in immunological and biochemical markers following treatment of AAV.***

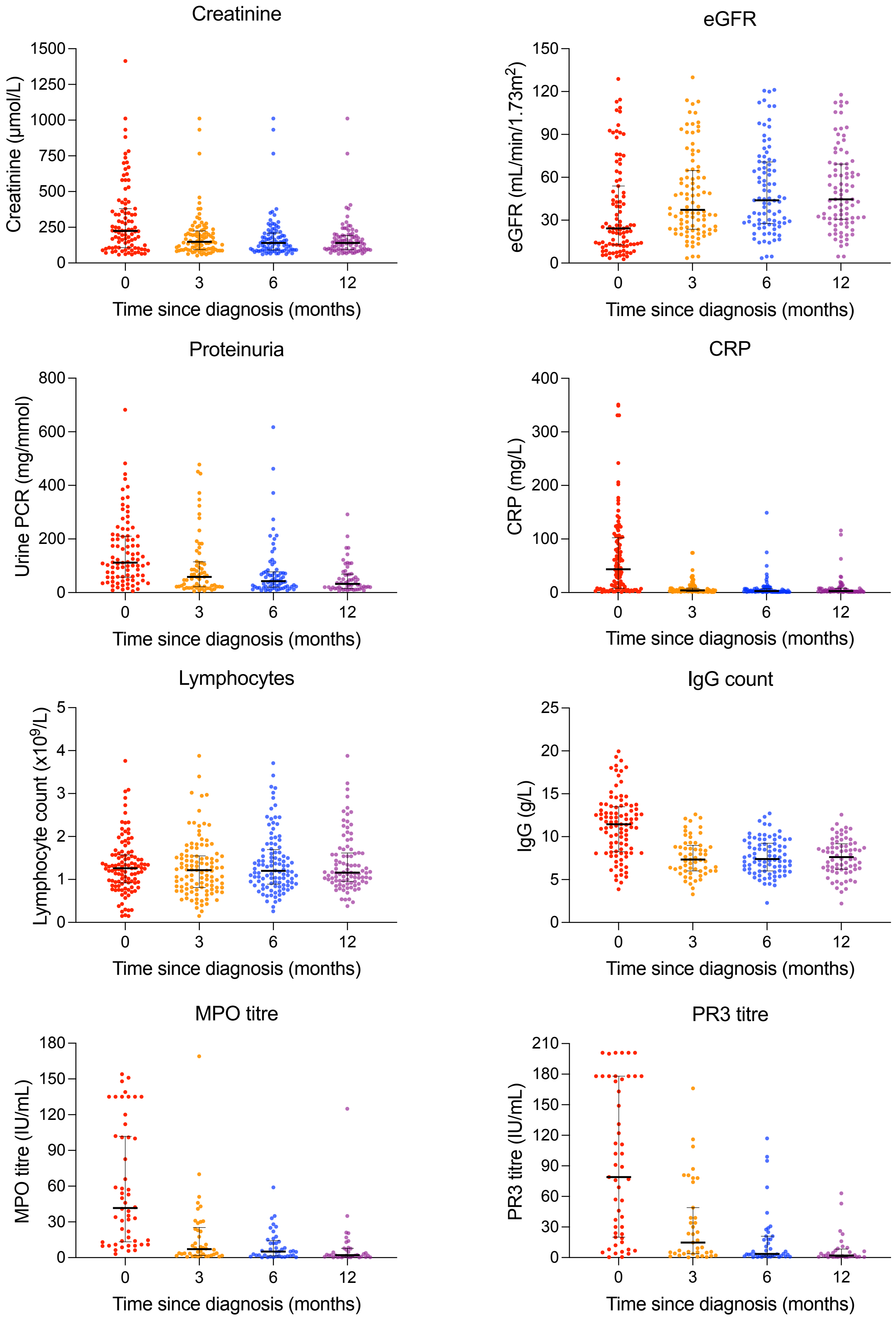

Supplementary figure S4. ***Oral glucocorticoid therapy and markers of disease severity.*** Scatter plots of oral glucocorticoid duration and dose stratified by baseline kidney function **(A & B)**, proteinuria **(C & D)**, and index kidney biopsy findings categorised by Berden classification **(E & F)** and ANCA renal risk score **(G & H)**.

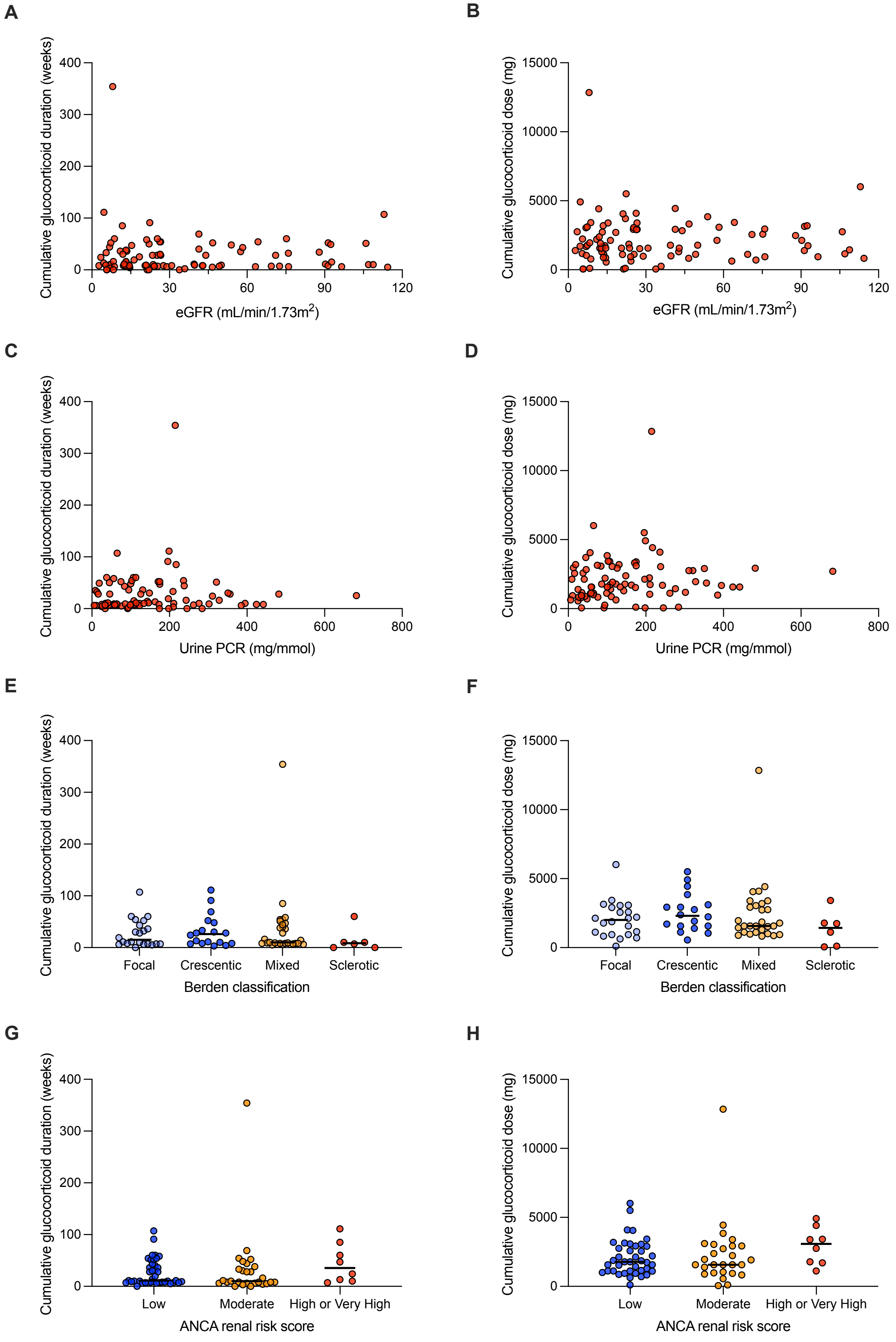

STROBE checklist

Based on the STROBE guidelines.

n/a: not applicable

Section/Topic	Item	Checklist item	Page, Table, Figure
**Title and abstract**			
Title	1a	Indicate the study's design with a commonly used term in the title or abstract	Page 2
Abstract	1b	Provide in the abstract an informative and balanced summary of what was done and what was found	Pages 3
**Introduction**			
Background/rationale	2	Explain the scientific background and rationale for the investigation being reported	Pages 5-6
Objectives	3	State specific objectives, including any prespecified hypotheses	Page 6
**Methods**			
Study design	4	Present key elements of study design early in the paper	Page 7-9
Setting	5	Describe the setting, locations, and relevant dates, including periods of recruitment, exposure, follow-up, and data collection	Pages 7-9
Eligibility criteria	6a	Give the eligibility criteria, and the sources and methods of selection of participants. Describe methods of follow-up	Page 7-9
	6b	For matched studies, give matching criteria and number of exposed and unexposed	n/a
Variables	7	Clearly define all outcomes, exposures, predictors, potential confounders, and effect modifiers. Give diagnostic criteria, if applicable	Pages 7-9
Data sources / management	8	For each variable of interest, give sources of data and details of methods of assessment. Describe comparability of assessment methods if there is more than one group. Give information separately for exposed and unexposed groups, if applicable	Pages 7-9
Bias	9	Describe any efforts to address potential sources of bias	Page 7-9
Study size	10	Explain how the study size was arrived at	Page 7
Quantitative variables	11	Explain how quantitative variables were handled in the analyses. If applicable, describe which groupings were chosen, and why	Page 7-9, page 12
Statistical methods	12a	Describe all statistical methods, including those used to control for confounding	Page 9
	12b	Describe any methods used to examine subgroups and interactions	Page 9
	12c	Explain how missing data were addressed	Page 9
	12d	If applicable, explain how loss to follow-up was addressed	n/a
	12e	Describe any sensitivity analyses	n/a
**Results**			
Participants	13a	Report number of individuals at each stage of study (e.g., numbers potentially eligible, examined for eligibility, confirmed eligible, included in the study, completing follow-up, and analysed. Give information separately for exposed and unexposed groups if applicable	Page 10
	13b	Give reasons for non-participation at each stage	n/a
	13c	Consider use of a flow diagram	n/a
Descriptive data	14a	Give characteristics of study participants (e.g., demographic, clinical, social) and information on exposures and potential confounders. Give information separately for exposed and unexposed groups if applicable	Page 10, Tables 1 & 2<br>Supplementary Table 2
	14b	Indicate number of participants with missing data for each variable of interest	Tables 2 & 3
	14c	Summarise follow-up time (e.g., average and total amount)	Page 12
Outcome data	15	Report numbers of outcome events or summary measures over time. Give information separately for exposed and unexposed groups if applicable	Pages 11-13<br>Tables 2 & 3<br>Supplementary Tables 2 & 3
Main results	16a	Give unadjusted estimates and, if applicable, confounder-adjusted estimates and their precision (e.g., 95% confidence intervals). Make clear which confounders were adjusted for and why they were included	Pages 10-13
	16b	Report category boundaries when continuous variables were categorised	n/a
	16c	If relevant, consider translating estimates of relative risk into absolute risk for a meaningful time period	n/a
Other analyses	17	Report other analyses done – e.g., analyses of subgroups and interactions, and sensitivity analyses	n/a
**Discussion**			
Key results	18	Summarise key results with reference to study objectives	Page 14

Limitations	19	Discuss limitations of the study, taking into account sources of potential bias or imprecision. Discussion both direction and magnitude of any potential bias	Page 17
Interpretation	20	Give a cautious overall interpretation considering objectives, limitations, multiplicity of analyses, results from similar studies, and other relevant evidence	Page 16-17
Generalisability	21	Discuss the generalisability (external validity) of the study results	Page 14-17
**Other information**			
Funding	22	Give the source of funding and the role of the funders for the present study and, if applicable, for the original study on which the present article is based	n/a
